# Gender and home language effects on vocabulary skills among school children aged 9–15 in Finland

**DOI:** 10.1038/s41598-025-28902-w

**Published:** 2025-12-29

**Authors:** Raymond Bertram, Tomi Rautaoja, Santeri Holopainen, Tuomo Häikiö, Petra Enges, Jukka Hyönä, Minna Lehtonen, Kenneth R. Pugh, Jay G. Rueckl, Rosa Salmela, Noam Siegelman, Pekka Räsänen

**Affiliations:** 1 Department of Psychology and Speech-Language Pathology, University of Turku, Turku, Finland; 2 Turku Research Institute for Learning Analytics, University of Turku, Turku, Finland; 3 Department of Psychological Sciences, University of Connecticut, Storrs, USA; 4 Yale Reading Center, Yale School of Medicine, New Haven, USA; 5 Departments of Psychology and Cognitive and Brain Sciences, Hebrew University of Jerusalem, Jerusalem, Israel; 6 Epilepsia Helsinki, Department of Pediatric Neurology, HUS Helsinki University Hospital, Helsinki, Finland

**Keywords:** Vocabulary proficiency, Reading development, Gender gap, Home language, Language background, Finnish, Psychology and behaviour, Psychology, Risk factors

## Abstract

Vocabulary proficiency is a key predictor of reading development. However, vocabulary proficiency in school-age children is rarely assessed, especially in languages other than English. Moreover, because reading development differs depending on home language and gender, it is likely that these factors also influence the development of vocabulary proficiency. Here we report Finnish vocabulary proficiency of school-age children, examining its relationship with grade, gender, and home language. We utilize d-Lexize, a vocabulary test based on visual lexical decision, which we adapted from a previous test for adult L2 speakers. The test assesses vocabulary knowledge by accuracy and lexical retrieval speed through reaction time. Approximately 27,000 school children were tested in three experiments using different versions of d-Lexize. All experiments consistently show that vocabulary proficiency improves progressively from 3rd to 9th grade. The results also reveal an emerging gender gap: whereas girls perform equal to boys in the early stages, they exhibit a more extensive vocabulary and faster lexical retrieval in the later grades. Furthermore, the tests show that pupils from Finnish-only homes consistently outperform those from non-Finnish or mixed-language homes, with this gap widening over time. These results highlight the significance of language exposure and sociocultural factors during vocabulary development.

**Supplementary Information:**

The online version contains supplementary material available at 10.1038/s41598-025-28902-w.

## Introduction

Childhood is a time of rapid vocabulary growth. During their initial 6 years—that is, before they start to read—children have already accumulated a substantial vocabulary amounting to thousands of words. This progression continues steadily throughout the years of primary education. For German pupils, vocabulary grows from roughly 6000–38,000 words between 1st and 8th grade (age 6–14)^1^. The number of dictionary entries known by English-speaking Canadian children grows from about 10,000 to 40,000 from 1st to 5th grade^2^. There are, however, significant disparities in vocabulary size and growth rate among school children. Biemiller showed that by the end of 2nd grade, English-speaking children in the lowest 25th percentile know about 4000 root words, whereas those in the highest 25th percentile knew around 8000 root words^3^. Song et al. identified substantial individual differences in vocabulary size for Chinese children from 4 years onwards already^4^. Noticeable individual differences in early vocabulary skills have also been reported for Finnish^5,6^, although these studies had small sample sizes and limited grade coverage.

Vocabulary proficiency is at the heart of language proficiency. An extensive vocabulary is associated with good phonological skills^7^, syntactic advancement^8^, and well-developed listening and writing ability^9^. Importantly, vocabulary growth plays a crucial role in reading development, as children rely on their word knowledge to make sense of texts. Torppa et al. found that smaller early vocabulary size translates into slower progress in reading^10^. Conversely, Colenbrander et al. showed that children with poor reading comprehension typically exhibit relatively low vocabulary skills^11^. The relationship between vocabulary and reading comprehension is thus reciprocal. Children with smaller vocabularies tend to read less than their peers with larger vocabularies, which in turn reduces their exposure to new words.

Differences in vocabulary size may mediate the impact of certain sociolinguistic factors on the development of reading comprehension. For example, in the latest PISA tests, assessing pupils across 81 countries^12^, gender and home language emerged as prominent predictors of reading comprehension. Specifically, boys demonstrated lower reading skills than girls (see also^13,14^), and first- and second-generation immigrant children scored lower than their peers from families of native language speakers^12^. These effects of gender and home language on reading comprehension are found in all languages tested within PISA including Finnish. Given the strong relation between vocabulary knowledge and reading comprehension, similar effects may be expected for vocabulary development. Since PISA identifies these gaps at age 15, it is crucial to track them earlier in development to understand both how they emerge and evolve over time, as well as the potential factors that contribute to their formation.

The current study aims to track the gender and home language gap in vocabulary skills among children in Finland from the 3rd to the 9th grade, aged 9–15. In Finland, the 1st to 6th grade belong to primary education, while the 7th to 9th grade fall under lower secondary education. Both levels are compulsory for all children. Manu et al. found that while the gender gap in Finnish reading ability is negligible in the early stages of primary school education, it increases over time^15^. However, despite the early similarity in reading skills across genders, vocabulary differences may emerge in the early primary school years, potentially predicting later reading disparities. This study investigates that possibility. It also examines the emergence and development of the home language gap in Finnish vocabulary. Specifically, we compare children from fully Finnish-speaking homes with those from non-Finnish-speaking homes, as well as those from mixed homes where one caregiver is Finnish and the other is non-native. There is a clear need for this type of research, as there is limited understanding of how these three distinct home language environments shape vocabulary development until the end of lower secondary education when the PISA test is administered.

To accommodate this need, we developed d-Lexize (developmental Lexize), a comprehensive and reliable tool for assessing Finnish vocabulary skills in children from the 3rd to the 9th grade, aged 9–15 years. The d-Lexize test was derived from the Lexize vocabulary test for Finnish L2 speakers^16^. In turn, that test was modeled after the Lexical Test for Advanced Learners (LexTale), a validated test to assess vocabulary proficiency for adult English L2 learners^17^. Like LexTale, Lexize is based on a visual lexical decision task, wherein participants judge whether a visually presented letter string is a word (e.g., savory) or a pseudoword (e.g., plaudate). In other words, our focus was on print vocabulary knowledge rather than vocabulary knowledge in general; however, throughout the text, we refer to it simply as vocabulary knowledge. Another point is that the test taps into vocabulary breadth, meaning the number of words that are known or can be recognized, rather than vocabulary depth, which refers to how well these words are understood^18^. However, both dimensions of vocabulary knowledge not only correlate strongly with reading comprehension but also with each other^19^. In other words, when a person recognizes a large number of words, they typically also possess ample syntactic, semantic, and practical knowledge about them.

The Lexize test of Salmela et al. includes words that range from low to medium frequency, ensuring that it encompasses words of varying difficulty level^16^. Since Lexize detected differences among adult L1 and L2 Finnish speakers, we inferred that the test could serve as a starting point for designing a comprehensive Finnish vocabulary test for L1 and L2 school-aged children. This assertion is also supported by the Rapid Online Assessment of Reading ability test (ROAR) developed by Yeatman et al. for English^20^, which showed that visual lexical decision not only allows the assessment of adult vocabulary skills, but can be used equally well to tap into children’s vocabulary skills. Specifically, they created a simple web-based visual lexical decision task and showed that this can serve as an accurate and reliable measure of English reading ability (as assessed by the Woodcock-Johnson Word Identification test) from early childhood (6 years) onwards. An interesting finding of that study was that accuracy rate was a much better predictor of reading ability than reaction time. Yeatman et al. speculated that in languages with an opaque orthography, reaction time is not a reliable measure of individual differences, at least for young readers, but hypothesized that in transparent orthographies individual differences may be more distinctly reflected in reaction time^20^. They also noted that reaction time might work better with larger samples than the 100–200 participants used in their studies.

The current study employs a similar visual lexical decision in a transparent orthography, namely Finnish, in which each letter corresponds to a single phoneme and nearly every phoneme to a single letter (only the phoneme /ŋ/ as in *hanko* does not correspond to a unique letter but to ‘nk’ or ‘ng’). Moreover, by exploiting the nationwide educational platform ViLLE, to which 70% of all Finnish elementary and lower secondary schools are subscribed, we collected data from more than 27,000 pupils from the 3rd to the 9th grade. The transparent orthography is likely to ensure that both accuracy rate and reaction time can serve as reliable dependent measures, while the large sample size helps strengthen the generalizability of the findings. It is particularly valuable to use reaction time alongside accuracy rate, as this measure captures a different facet of vocabulary skills: accuracy rate reflects the number of words a participant knows, while reaction time more effectively captures the speed of lexical retrieval. Both aspects of vocabulary are essential skills that play distinct roles in reading fluency and reading comprehension. This is for instance shown in the ENRO (ENglish Reading Online) metastudy of Siegelman et al.^21^, who found through exploratory factor analyses that vocabulary accuracy and reaction time load on separate factors. Their results also showed that accuracy is closely linked to reading comprehension, while reaction time is more strongly associated with reading fluency.

The current study allows for several key questions to be explored. First, does vocabulary proficiency increase steadily throughout the school years? Second, is there a gender gap in the early stages of education already or does it emerge later? Third, how large is the vocabulary gap between children from Finnish and children from non-Finnish homes, and does this gap narrow over time? Fourth, do children from mixed homes have lower vocabulary proficiency than children from Finnish homes and if so, to what extent? While this study focuses on vocabulary development in Finnish, similar issues are relevant for other languages, as highlighted by the recent PISA results on reading comprehension, which show consistent effects of gender and home language across languages^12^.

The current study was conducted over three separate experiments, utilizing different versions of d-Lexize. Between experiments, the number of items was reduced based on Item Response Theory (IRT) analyses, driven by the goal of creating a more concise vocabulary test with the best possible items, while also considering the time constraints when testing school children. Each version of the d-Lexize task was tested for reliability and validated against a sentence reading proficiency test and a phonological word reading test (for specifications, see Methods section). The validity of the various d-Lexize versions was also assessed by analyzing the impact of word frequency and length on accuracy and reaction time, as these factors are known to influence reading and lexical decision^22,23^.

Experiment 1 tested approximately 7000 children from the 3rd, 4th, and 7th grade; the other experiments included approximately 5000 (Experiment 2) and 15,000 (Experiment 3) children from the 3rd to the 9th grade. Gender effects were assessed in all experiments, as the distribution of boys and girls was even across experiments. Home-language effects were assessed in Experiment 1 and 3 and involved 3 levels: children from exclusively Finnish-speaking homes (Finnish), children from mixed homes (Finnish/other), and children from non-Finnish-speaking homes (other). Information on the age of acquisition of the Finnish language was not available for the latter two categories, so the impact of home language was only assessed at a general level. Experiment 2 had too small a percentage of children from mixed and non-Finnish speaking households to consider home language as a variable.

Data on accuracy rate and reaction times were analyzed using (generalized) linear mixed-effects models with the lme4 package^24^ in R statistical software (Version 4.3.0^25^). Random intercepts were included for both participants and items^26^. We used GLMM to analyze accuracy rate and LMM for reaction times (RTs). The RTs that were computed were based on RTs for correct responses only. Due to skewness, RTs were log-transformed prior to analysis. We examined models where grade interacted with 3 or 4 variables: gender, home language (not in Experiment 2), word frequency, and word length with list as a control variable in Experiment 2 and 3 (as here we used two versions of d-Lexize). The analyses only included word data and not pseudoword data because words were our primary target of interest; moreover, the model includes Log Lemma Frequency as a predictor, a variable that is not available for pseudowords.

In Experiment 1, including the 3rd, 4th, and 7th grade, grade was treated as a categorical variable due to discontinuity between the grades, while in Experiments 2 and 3, with all grades from 3rd to 9th included, it was treated as a numeric variable. Gender and home language were also entered as categorical variables. The categorical variables were dummy coded with 3rd grade, boys and Finnish as the reference categories, respectively. Significance of the full terms was assessed by Wald tests (χ^2^) for accuracy and F-tests using the Satterthwaite approximation for the effective degrees of freedom for reaction times. For Experiment 1, post-hoc analyses were performed assessing the effect of gender and home language at each grade level with *p* values being adjusted for False Discovery Rate^27^. To account for potential variation in vocabulary performance across schools, we conducted a second analysis of Experiment 1, adding school as a random intercept. As this did not impact the results by any means, we only report the initial analyses in the running text. All full models and post-hoc analyses (including the extra analyses of Experiment 1) can be found in the statistical reports at https://osf.io/exb9s/?view_only=04dda2e11463493a8cc4bc76261d5e9c.

## Results

### Experiment 1

The Lexize task for adult L2 speakers included 68 words and 34 pseudowords, all of which were presented to the participants of Experiment 1. Due to the concern that some of these items might not be suitable for school children, we conducted an IRT analysis and identified 9 words and 4 pseudowords with poor psychometric properties (see Methods for further details). These items were excluded from subsequent analyses, resulting in a final set of 89 items, which we named d-Lexize89; the analyses in Experiment 1 are based on this set.

**Fig. 1:**
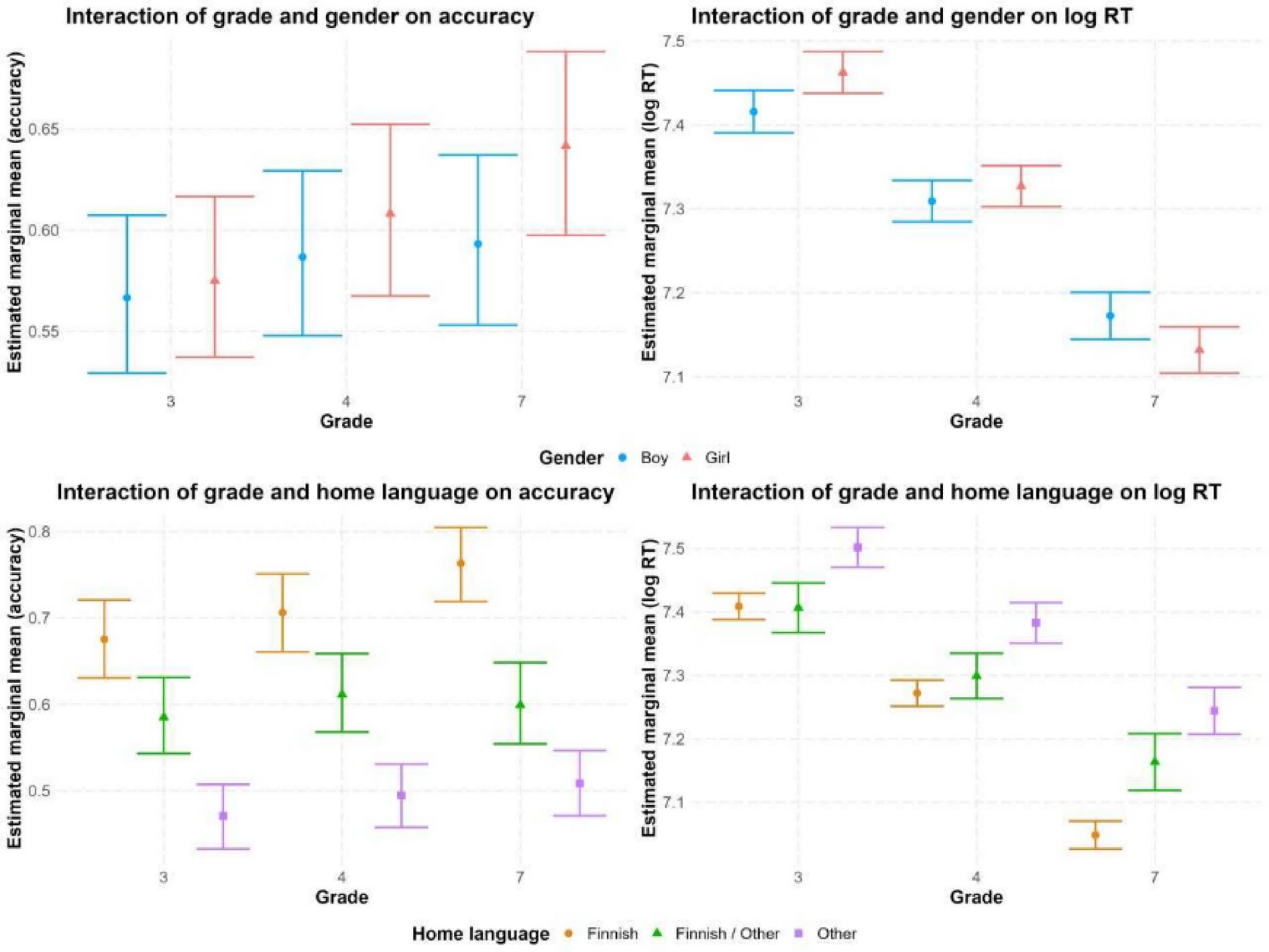
Mixed model-based marginal means for accuracy rates and RTs for words of d-Lexize89 for the interactions of grade with gender and home language.

In the analysis of accuracy rates, the main effects of grade (χ^2^(2) = 31.53, *p* < 0.001), home language χ^2^(2) = (686.5, *p* < 0.001), and the interactions between grade and gender (χ^2^(2) = 16.04, *p* < 0.001) as well as between grade and home language (χ^2^(4) = 25.17, *p* < 0.001) were significant (see Fig. 1). Post-hoc comparisons revealed a growing gender difference in vocabulary proficiency from 3rd to 7th grade: While no significant difference was found in grade 3 (OR = 0.942, SE = 0.038, Z = − 1.502, *p* = 0.142), boys scored significantly lower than girls in grade 4 (OR = 0.866, SE = 0.034, Z = − 3.639, *p* = 0.001). The advantage for girls had even become larger by grade 7 (OR = 0.732, SE = 0.036, Z = − 6.387, *p* < 0.001). For home language, post-hoc analyses showed that already in grade 3, children from Finnish-speaking homes outperformed those from mixed-language homes (OR = 1.773, SE = 0.13, Z = 7.83, *p* < 0.001) and non-Finnish homes (OR = 4.238, SE = 0.237, Z = 25.81, *p* < 0.001). In turn, children from mixed-language homes had a higher accuracy rate than those from non-Finnish homes (OR = 2.391, SE = 0.206, Z = 10.12, *p* < 0.001). Similar patterns were observed in grade 4 (Finnish vs. Mixed: OR = 1.789, SE = 0.118, Z = 8.809, *p* < 0.001; Finnish vs. Other: OR = 4.237, SE = 0.244, Z = 25.08, *p* < 0.001; Mixed vs. Other: OR = 2.368, SE = 0.193, Z = 10.57, *p* < 0.001). By grade 7, the gap had widened between children from Finnish-speaking homes, maintaining the highest accuracy rates, and children from mixed and non-Finnish homes (Finnish vs. Mixed: OR = 2.758, SE = 0.239, Z = 11.719, *p* < 0.001; Finnish vs. Other: OR = 5.386, SE = 0.377, Z = 24.05, *p* < 0.001). There was also still a clear difference between children from mixed-language and non-Finnish homes (Mixed vs. Other: OR = 1.953, SE = 0.203, Z = 6.44, *p* < 0.001).

For RT, there were again significant effects for grade (F(2, 25,035) = 52.31, *p* < 0.001) and home language (F(2, 7041) = 106.99, *p* < 0.001) as well as interactions between grade and gender (F(2, 6811) = 14.77, *p* < 0.001), and grade and home language, F(4, 7042) = 8.40, *p* < 0.001. Post-hoc comparisons revealed a shift in the gender effect across grades: in grade 3, boys responded significantly faster than girls (β = − 0.047, SE = 0.01, Z = − 4.541, *p* < 0.001), but by grade 4, there was no difference (β = − 0.018, SE = 0.01, Z = − 1.746, *p* = 0.081), and by grade 7, the pattern had reversed, with boys now responding significantly slower than girls (β = 0.041, SE = 0.012, Z = 3.269, *p* = 0.001). For home language, post-hoc comparisons showed that in grade 3, children from Finnish-speaking or mixed homes responded significantly faster than children from non-Finnish-speaking homes (Finnish vs. Other: β = -0.093, SE = 0.015, Z = − 6.379, *p* < 0.001; Mixed vs. Other: β = − 0.095, SE = 0.022, Z = − 4.246, *p* < 0.001), whereas the difference between fully Finnish and mixed language homes was not significant (Finnish vs. Mixed: β = 0.002, SE = 0.019, Z = 0.112, *p* = 0.91). The same pattern was found for the 4^th^ grade: (Finnish vs. Other: β = − 0.111, SE = 0.015, Z = − 7.409, *p* < 0.001; Mixed vs. Other: β = − 0.084, SE = 0.021, Z = − 3.951, *p* < 0.001; Finnish vs. Mixed: β = − 0.027, SE = 0.017, Z = − 1.596, *p* = 0.121). In grade 7, all home language contrasts reached significance (Finnish vs. Mixed: β = − 0.115, SE = 0.022, Z = − 5.168, *p* < 0.001, Finnish vs. Other: β = − 0.196, SE = 0.018, Z = − 10.788, *p* < 0.001; Mixed vs. Other: β = − 0.081, SE = 0.027, Z = − 2.992, *p* = 0.003). Similar to the findings for accuracy, the gap between children from Finnish and non-Finnish homes had also widened for RTs, reflecting an increasing disparity in lexical retrieval speed. Moreover, children from Finnish homes now responded faster than those from mixed-language homes, suggesting that a gap in lexical retrieval speed is also emerging between these groups across grades. Figure 1 shows the interactions of grade with gender and home language for accuracy and RT in Experiment 1.

### Experiment 2

In Experiment 2 we sought to reduce the length of the d-Lexize test and to create an additional version of the test, allowing for multiple assessments and the potential development of a parallel assessment of oral vocabulary. Therefore, from the 89 items of d-Lexize89, we created two item lists of 36 words and 19 pseudowords (d-Lexize55a & d-Lexize55b), each containing 34 unique items and 21 shared items (for more detailed information, see Methods). The lists were matched on average item discriminability and accuracy rate in Experiment 1 as well as on average word and bigram frequency (i.e., the average frequency of all adjacent letter pairs in a word), word length, and orthographic neighborhood; list was entered as a control variable in the analyses.

**Fig. 2:**
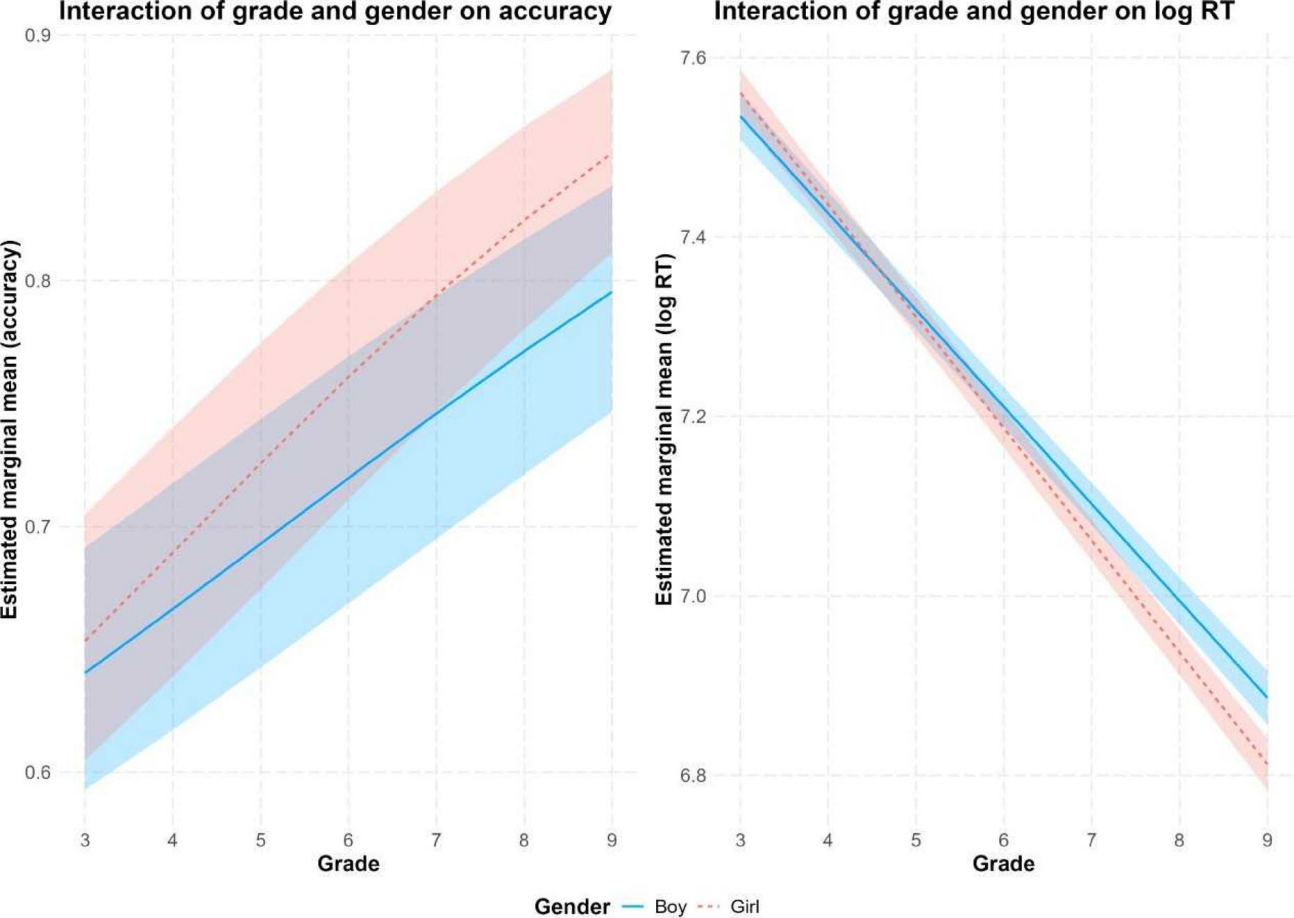
Mixed model-based marginal means for accuracy rates and RT for words of d-Lexize55 for the interaction of grade with gender.

For accuracy, the analysis of Experiment 2 showed a marginal effect for grade (χ^2^(1) = 3.57, *p* = 0.059) and no effect for gender (χ^2^(1) = 1.05, *p* = 0.31), but the grade by gender interaction was significant (χ^2^(1) = 14.07, *p* < 0.001). As in Experiment 1, this indicates that the initially similar performance in early grades shifts to girls outperforming boys in later grades (see Fig. 2, left panel). For RT, significant effects were observed for grade (F(1, 55,421) = 40.97, *p* < 0.001), gender (F(1, 5185) = 10.06, *p* = 0.002), and the grade by gender interaction (F(1, 5161) = 16.55, *p* < 0.001). This interaction is in line with the RT results in Experiment 1 and indicates a shift from boys being faster in grade 3 to girls being faster in the later grades (see Fig. 2, right panel). The effect of list was not significant (Accuracy, *p* = 0.98; RT, *p* = 0.08). Figure 2 shows the interaction of grade with gender for accuracy and RT in Experiment 2.

### Experiment 3

IRT analyses of Experiment 2 identified 10 words with poor psychometric properties (see Methods for more details). These items were excluded from Experiment 3. From the remaining 79 items, we constructed two lists with 25 words and 16 pseudowords (d-Lexize41a & d-Lexize41B), each of which had 38 unique items and 3 shared items. The lists were matched on average item discriminability and item accuracy from Experiment 2 as well as on average word and bigram frequency, word length, and orthographic neighborhood.

**Fig. 3:**
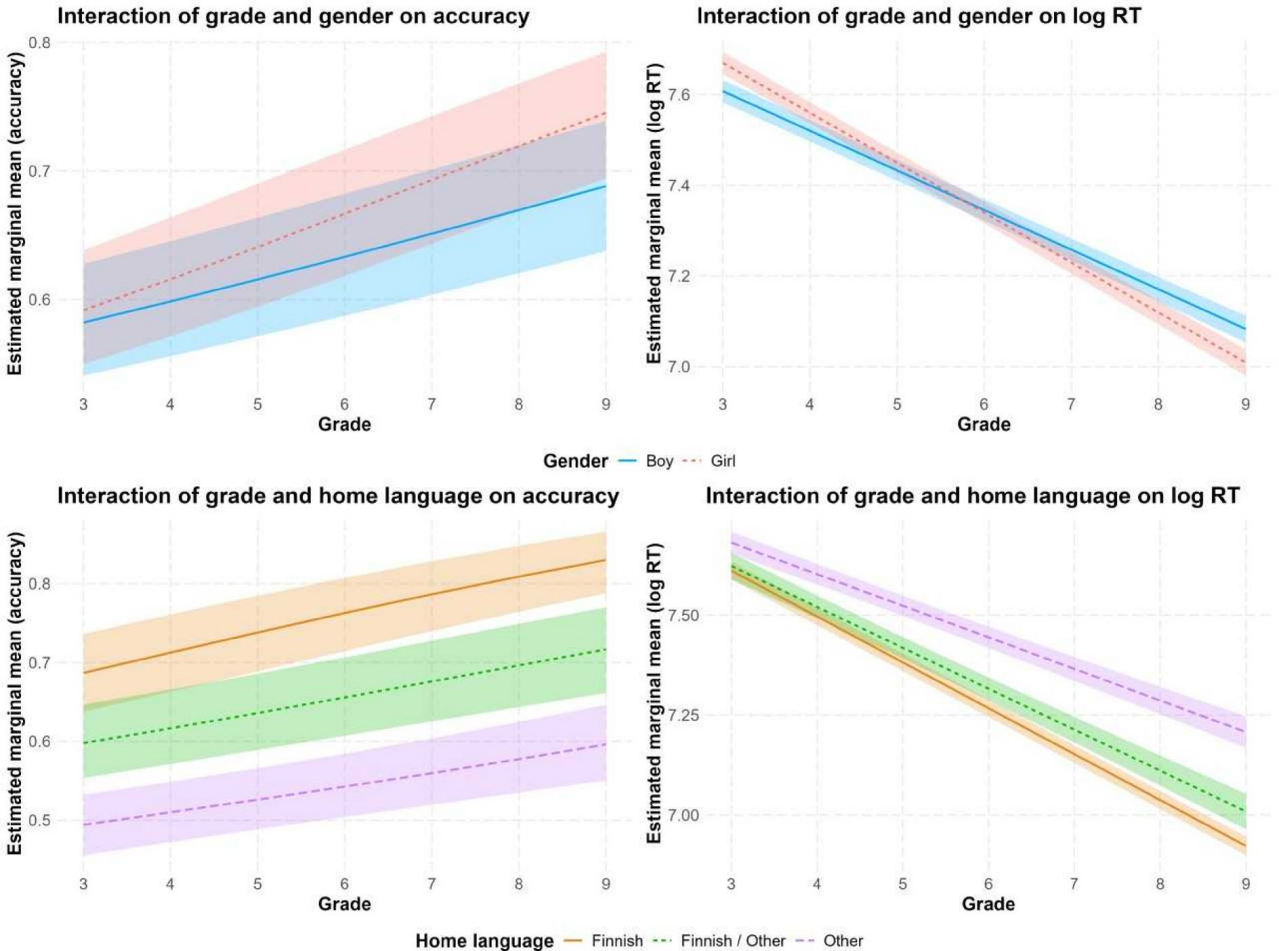
Mixed model-based marginal means for accuracy rates and RT for words of d-Lexize41 for the interactions of grade with gender and home language.

For accuracy, there was a significant main effect for grade (χ^2^(1) = 57.31, *p* < 0.001), but not for gender (χ^2^(1) = 2.14, *p* = 0.14). The interaction between grade and gender was again significant (χ^2^(1) = 25.55, *p* < 0.001), indicating that similar performance in earlier grades turned into an advantage for girls in later grades. There was also a main effect for home language (χ^2^(2) = 296.02, *p* < 0.001), indicating that children from exclusively Finnish homes outperformed children from mixed homes (OR = 0.63, CI 0.52–0.77, *p* < 0.001) and children from non-Finnish homes (OR = 0.63, 95% CI 0.25–0.33, *p* < 0.001). The significant interaction between grade and home language (χ^2^(2) = 6.05, *p* = 0.049) reflected that the gap between children from exclusively Finnish homes with children from mixed homes (OR = 0.97, CI 0.93–1.00, *p* = 0.076) and non-Finnish homes (OR = 0.97, CI 0.95–1.00, *p* = 0.061) is growing over the years. The effect of list was not significant (χ^2^(1) = 3.82, *p* = 0.051). The interactions between grade and gender and grade and home language are depicted in Fig. 3 (left panels).

For RT, there was a significant main effect for grade (F(1, 77,857) = 387.78, *p* < 0.001) and gender (F(1, 14,562) = 98.32, *p* < 0.001) and the interaction between grade and gender was also significant (F(1, 14,375) = 90.89, *p* < 0.001). The interaction indicated that the initial faster responses for boys in the early grades swapped towards faster responses for the girls in the later grades. There was no main effect for home language (F(2, 15,623) = 2.09, *p* = 0.12), but the interaction between grade and home language was again significant (F(2, 15,033) = 44.30, *p* < 0.001). This indicated that the gap between children from Finnish-speaking homes and those from mixed-language homes widens over the school years (β = 0.01, 95% CI 0.00–0.02, *p* = 0.01) and grows even more in relation to children from non-Finnish-speaking homes (β = 0.04, 95% CI 0.03–0.04, *p* < 0.001). The effect of list was not significant (F(2, 33,497) = 0.62, *p* = 0.43). Figure 3 shows the interactions of grade with gender and home language for accuracy and RT in Experiment 3.

## General discussion

The results from our large-scale study provide clear and consistent answers to the questions posed in the Introduction, replicated across three versions of the d-Lexize vocabulary test, all targeting print vocabulary, and across three different samples. First, as expected, it became clear that vocabulary proficiency increases steadily throughout the school years. This is reflected in increasing accuracy demonstrating vocabulary growth from 3rd to 9th grade, in line with earlier studies showing a steady increase of vocabulary during the school years in German^1^ and English^2^. Increasing vocabulary proficiency is also reflected by the progressively faster reaction times, indicating increasing speed of lexical retrieval, in line with studies that show a solid increase in reading speed throughout the school years^28^.

Second, a gender gap was observed in vocabulary skills, similar to what has been reported for reading comprehension, where girls tend to outperform boys^13,14^. Importantly, however, this gap was not evident in the early school years. Specifically, across all Experiments, 3rd grade boys appeared to be on par with 3rd grade girls in vocabulary knowledge and even demonstrated faster lexical retrieval. By 7th grade, however, around the transition from primary to lower secondary school, girls had surpassed boys in both vocabulary size and retrieval speed. This suggests that boys’ weaker vocabulary skills do not clearly precede their weaker reading comprehension, as speculated in the Introduction. Instead, vocabulary difficulties appear to align with the developmental trajectory of reading comprehension difficulties^15^. The gender gap in reading comprehension is often attributed to differences in reading motivation and habits, with girls typically displaying greater intrinsic reading motivation and reading more frequently than boys^29,30^. This difference becomes even more evident by the end of elementary school, around 6th grade^31^. It is likely that the observed disparity and development in vocabulary skills stems from similar underlying factors.

Third, not speaking Finnish at home affects vocabulary proficiency greatly. Children from non-Finnish homes have a smaller vocabulary and are slower in lexical retrieval than children from exclusively Finnish or mixed homes, which is in line with the most recent PISA results^12^. These differences seem to be present throughout the school years, and are even more pronounced in the later grades than in the earlier grades. Since we did not collect information on the age at which children acquired Finnish, we cannot determine whether this widening gap is linked to students in higher grades having spent less time in Finland than those in lower grades. This is something we plan to explore further in future testing rounds. It is nevertheless likely that our results reflect that the school environment and education cannot effectively bridge the vocabulary and reading comprehension gap during the school years, which, in an ideal scenario, they would. However, this may be a lot to expect when in the language environment outside school exposure to Finnish is relatively limited or of lower quality. Parental support for developing Finnish proficiency in non-Finnish homes may for instance be limited because families prioritize maintaining their heritage language at home or because they are not proficient in Finnish themselves.

Fourth, having another home language in addition to Finnish turns out to affect vocabulary proficiency as well, even though the gap is smaller compared to children from non-Finnish homes. However, the results are evident in both vocabulary knowledge and lexical retrieval. These results align with other studies indicating that simultaneous bilingualism can limit proficiency in the dominant language due to less frequent exposure and use of words. For instance, Bialystok et al. reported the results of an analysis of 1738 children between 3 and 10 years old and demonstrated a consistent difference in receptive vocabulary between monolinguals and bilinguals^32^. This aligns with the notion that bilingual children in early childhood receive less exposure to the majority language at home than monolingual children. As a result, they encounter fewer words, leading to lower accuracy scores, and have less repeated exposure to the words they do learn, contributing to slower lexical retrieval. Perhaps surprisingly though, our results show that the vocabulary gap widens as children progress through school. One might expect children from mixed-language homes to catch up over time, as their daily exposure to Finnish increases in elementary school—not only through spoken interactions but also through written language. To gain a deeper understanding of the widening gap between monolingual and bilingual pupils, future studies should include more detailed questionnaires on language use of bilingual children at home and elsewhere and explore their relationship to vocabulary development.

## Conclusions and future directions

The current study assessing print vocabulary skills revealed differences based on grade level, gender, and home language. Since vocabulary proficiency develops across the school years and disparities emerge gradually, monitoring it from early education onwards would be essential. However, this kind of monitoring is rarely implemented in school assessment practices, partly due to the limited availability of standardized vocabulary assessments in many languages, including Finnish. The present study addresses this need by developing d-Lexize, a valid and reliable instrument for assessing vocabulary proficiency. The next step in this process involves creating normative data for 1st to 9th grade, a task we are currently undertaking. Expanding access to reliable vocabulary measures would support early identification of difficulties and enable targeted vocabulary interventions.

The current study is part of a larger initiative entitled the Multilingual Reading Assessment (MUREA) project. Future research within the MUREA framework aims to comprehensively investigate the development and remediation of components of reading comprehension in individuals from diverse linguistic backgrounds. In addition to vocabulary, this includes phonology, morphology, syntax, and self-regulation skills. As such, the project aims to contribute to both reading research and practical applications in educational settings, ultimately promoting more equitable language development across diverse learner populations.

## Methods

For each Experiment, the R code as well as R-generated HTML-reports are available at the project’s OSF page, https://osf.io/exb9s/?view_only=04dda2e11463493a8cc4bc76261d5e9c. The HTML-reports include the participant selection procedure, IRT analyses, validity and reliability tests, Monte Carlo simulations, (g)lmm models, and post-hoc analyses as well as outlier removal, including participant exclusions and filtering of extreme reaction times.

### Participants

The children were sampled from children from schools across the regions of Uusimaa and Southwest Finland. Our dataset comes from municipal registries which focuses on monitoring school performance. These registries do not include information like place of birth or immigration year, but do include home language as a language-related variable. The assessments were performed in mainstream classrooms and are used to monitor learning and guide support. There was no specific diagnostic information on reading or writing difficulties collected or available. Across experiments, around 27,000 participants were included in the analyses, with some excluded beforehand (see HTML reports for details). From all the children included in the analyses informed parental consent was obtained. A total of 6,988 children (50% boys, 50% girls; home language: 77% Finnish; 14% Other; 9% Finnish/Other) from the 3rd (n = 2,607), 4th (n = 2,691), and 7th grade (n = 1,784) were included in the analyses of Experiment 1. In the analyses of Experiment 2, 5,205 children (52% girls, 48% boys) with home language Finnish from the 3rd to the 9th grade (3rd: 862; 4th: 880; 5th: 877; 6th: 957; 7th: 726; 8th: 540; and 9th: 363) were included. Here children from mixed or non-native homes were excluded, as their percentage was very low in this sample (about 3%); hence, home language was not used as a variable in the analyses. For Experiment 3, the analyses included 14,634 children (50% girls, 50% boys) from the 3rd to the 9th grade (3rd: 5,108; 4th: 1,577; 5th: 1,789; 6th: 1,341; 7th: 3,301; 8th: 949; and 9th: 776). Here home language was used as a variable again, as there was a sufficient number of children from mixed (n = 1110, 7.6%) and non-Finnish homes (n = 1803, 12.3%). The proportion of children from non-Finnish homes in the current study (Experiment 1, 14%, Experiment 3, 12%) was comparable to the proportion of children with foreign backgrounds at the end of 2024 aged 0–14 in Finland (115,415 out of 820,287 children, about 14%)^33^.

### Procedure

Before the experimental tests, participants were administered a questionnaire including questions about gender and home languages. After this, participants completed the d-Lexize task and two tasks that were used for validation: the sentence reading fluency task (SRF; Experiments 1–3) and the phonological word reading task (PWR; Experiments 2 and 3). Prior to starting d-Lexize, participants were presented with instructions explaining that letter strings would be displayed one by one and that they were to indicate whether each letter string was a word or not by pressing the “yes” or “no” button. After the instructions, participants were presented with 4 or 5 practice items, depending on the experiment. Each letter string was preceded by a fixation point that remained on the screen for 1000 ms. There was a time limit for the individual items of 4000 ms in Experiment 1 and 2 and 5000 ms in Experiment 3. The d-Lexize test lasted 5–10 min, the whole battery between 25 and 40 min.

### The sentence reading fluency test (SFR)

In the SFR, participants read a series of sentences and are asked to indicate whether each sentence is true or not. The task is based on the widely used Woodcock-Johnson IV (WJ IV) sentence reading fluency test in English^34^. The current Finnish test includes four practice stimuli, followed by feedback after each response. After completing the practice items, each participant progresses through the task with as many stimuli as can be processed within the specified time limit (1 min and 30 s). A maximum of 41 sentences are presented, in fixed order. Statements are relatively easy to respond to (e.g., “stones can be eaten”, “cows fly”, “fishes swim”), so they elicit a high accuracy rate. However, how quickly the sentences are read varies considerably across grades and across children. The internal consistency for the test is excellent for reaction times and moderate for accuracy rate (Guttman’s lambdas were 0.94 and 0.74, respectively).

### The phonological word reading test (PWR)

In the PWR, participants are tasked with choosing a word that matches a presented picture. The task includes four practice stimuli, followed by feedback after each response. After completing the practice items, each participant progresses through the task with as many stimuli as can be processed within the specified time limit (2 min). A maximum total of 140 trials are presented in fixed order. In addition to the correct word, participants are presented with three foils, including words and nonwords, that vary in phonological—and, by extension, orthographic—similarity to the correct choice. In this task, word level reading is evaluated by assessing the participant’s ability to read and determine the word corresponding to the picture accurately. The internal consistency of the test is excellent for reaction times (Guttman’s lambda = 0.92) and good for accuracy rate (Guttman’s lambda = 0.83).

### The d-Lexize vocabulary test

In Experiment 1, we utilized the Lexize version developed by Salmela et al.^16^, comprising 102 items, with 68 being Finnish words and 34 phonotactically valid Finnish pseudowords. Lexical-statistical characteristics were extracted from a Finnish newspaper corpus containing 22.7 million word forms, utilizing the lexical search program WordMill^35^. The chosen words were drawn from six distinct frequency ranges: 17 words with frequency < 1 per million (pm); 20 words with frequency 1–5 pm; 19 words with frequency 5–10 pm; 11 words with frequency 10–20 pm; 1 word with frequency 20–100 pm. The word set predominantly consisted of nouns (n = 52), with a smaller representation of verbs (n = 7) and adjectives (n = 9), reflecting the proportion of these word classes in natural language. To avoid morphological structure aiding word recognition, all selected words were monomorphemic^36^. Word length varied from 4 to 9 letters (M = 5.8). The set of 34 pseudowords was derived from words that were matched in part of speech, length, and frequency with the 68 word items. In these chosen words, 1 to 3 letters were altered such that phonotactically valid pseudowords were created. The mean bigram frequency of the pseudowords (M = 5.9, SD = 2.5) aligned with that of the selected words (M = 6.1, SD = 2.6), as confirmed by an independent samples t-test (t(100) = 0.53, *p* = 0.60). This ensured that the letter patterns of the pseudowords mimic those in words. Subsequent versions of d-Lexize, i.e. d-Lexize89, d-Lexize55a, d-Lexize55b, d-Lexize41a, and d-Lexize41b, preserved the same properties as the original version. The characteristics of each d-Lexize version are listed in Table 1. All items from all versions are listed in Supplementary Table 1.

**Table 1:** Properties of the different Lexize versions.

Version	N	W^a^	PW^b^	Nom^c^	Adj^d^	V^e^	Freq^f^	LenW^g^	LenPW^h^	BiW^i^	BiPW^j^
Lexize	102	68	34	49	12	7	5.6 (0.1–26.2)	5.8 (4–9)	6.1 (5–9)	6.2	5.9
d-Lexize89	89	59	30	43	9	7	6.0 (0.1–26.2)	5.8 (4–9)	6.2 (5–9)	6.2	6.2
d-Lexize55a	55	36	19	26	6	4	5.9 (0.1–18.8)	5.9 (4–9)	6.3 (5–9)	6.0	6.2
d-Lexize55b	55	36	19	28	5	3	6.1 (0.1–26.2)	5.9 (4–9)	6.2 (5–9)	6.3	6.2
d-Lexize41a	41	26	15	18	5	3	5.9 (0.1–18.8)	6.0 (4–9)	6.3 (5–9)	6.4	6.2
d-Lexize41b	41	26	15	19	5	2	6.1 (0.1–26.2)	5.6 (4–7)	6.2 (5–9)	5.7	6.2

^a^No. of Words; ^b^No. of Pseudowords; ^c^No. of Nouns; ^d^No. of Adjectives; ^e^No. of Verbs; f. Frequency per million (range); ^g^Length of words (range); ^h^Length of pseudowords (range); ^i^Bigram Frequency of Words per 1000; ^j^Bigram Frequency of Pseudowords per 1000.

### From Lexize to d-Lexize89 to d-Lexize55 to d-Lexize41 via IRT and MC simulations

Experiment 1 started with the 68 words and 34 pseudowords from the Lexize test originally designed for L2 adults^16^. Given the possibility that some of these items may not be appropriate for a vocabulary test for school children, we conducted Item Response Theory (IRT) analyses on the combined data from the 3rd, 4th, and the 7th grade using the one-parameter (1PL), two-parameter (2PL), and three-parameter (3PL) logistic models, which account for item difficulty, discrimination, and guessing, respectively. The 3PL model provided the best fit statistics (see Sect. 2.4.4 from the html-report on Experiment 1 to be found at https://osf.io/exb9s/files?view_only=04dda2e11463493a8cc4bc76261d5e9c) and identified 9 words (itara ‘stingy’, kovera ‘concave’, houre ‘phantom’, uuhi ‘ewe’, kieppi ‘coil’, vouti ‘magistrate’, aihio ‘work in progress’, purje ‘sail’ and suppea ‘narrow’) and 4 pseudowords with poor psychometric properties, which either meant relatively low discriminability values, relatively low difficulty values, or close-to-zero guessing values. The exclusion of these items resulted in a final set of 89 items, which we named d-Lexize89; the analyses in Experiment 1 are based on this set. Next we conducted a Monte Carlo simulation on **d-Lexize89** with 17 sub-samples (sizes 5–85 in steps of 5). Table 3.4 in the HTML report of Experiment 1 to be found at https://osf.io/exb9s/files?view_only=04dda2e11463493a8cc4bc76261d5e9c shows mean correlations across 1000 repetitions. A 55-item sub-sample correlated almost perfectly (0.96 for accuracy, 0.99 for reaction times) with the full scale, so we used this number of items for the lists we created in Experiment 2.

*Experiment 2* split d-Lexize89 into two lists (**d-Lexize55a & d-Lexize55b**), each with 36 words and 19 pseudowords (34 unique and 21 shared). Lists were matched on item discriminability and accuracy derived from the IRT analyses in Experiment 1, as well as on average frequency, word length, bigram frequency, and orthographic neighborhood (see Table 1). The division into two lists of 55 items with equal lexical-statistical properties was made with future experimentation in mind, such as multiple testing and comparing vocabulary skills through parallel auditory and visual lexical decision tasks. Next, we again conducted a Monte Carlo simulation on **d-Lexize55a and d-Lexize55b** with 10 sub-samples (sizes 5–50 in steps of 5). The table in Sect. “Conclusions and future directions” of the HTML-report on Experiment 2 shows mean correlations across 1000 repetitions. A 40-item sub-sample correlated almost perfectly (0.96 for accuracy, 0.99 for reaction times, for both lists) with the full scale, so we used this number of items for the lists we created for Experiment 3. IRT analyses combining data from the 3rd to the 9th grades detected the 10 words with the least favorable psychometric properties and excluded them for Experiment 3. The 10 excluded items were: juhta ‘beast of burden’, kolttu ‘old-fashioned dress’, rahvas ‘common people’, pisara ‘drop’, kohtu ‘uterus’, tyrkyttää ‘intrude’, parvi ‘loft’, nuotio ‘campfire’, hauki ‘pike’ and sukeltaa ‘dive’.

*Experiment 3* further refined d-Lexize89 by excluding 10 words based on Experiment 2’s IRT analyses. The remaining 79 items were split into two lists (**d-Lexize41a & d-Lexize41b**), each containing 26 words and 15 pseudowords—38 unique and 3 shared—again matched for key linguistic properties (see Table 1).

### Reliability estimates for d-Lexize

We calculated Cronbach’s alpha and Guttman’s lambda 4 reliability coefficients for each class separately and for the total sample in each of the d-Lexize versions. We report Cronbach’s alpha due to its widespread use and familiarity, but also included Guttman’s lambda 4, which provides a more accurate estimate of test reliability by using the best possible split (Revelle and Condon, 2019)^37^. Here, we report the values for the total sample; however, the results for each grade were similar (for more details, see the internal consistency sections in each experiment’s HTML report). For d-Lexize89, Cronbach’s alpha was 0.90 for accuracy and 0.98 for RT, with Guttman’s lambda at 0.92 and 0.98, respectively. For d-Lexize-55a, Cronbach’s alpha was 0.78 for accuracy and 0.98 for latencies, and Guttman’s lambda was 0.82 and 0.98, respectively. For d-Lexize-55b, Cronbach’s alpha was 0.79 for accuracy and 0.98 for latencies, with Guttman’s lambda at 0.83 and 0.98, respectively. For d-Lexize-41a, Cronbach’s alpha was 0.80 for accuracy and 0.97 for latencies, and Guttman’s lambda was 0.84 and 0.98, respectively. For d-Lexize-41b, Cronbach’s alpha was 0.83 for accuracy and 0.97 for latencies, with Guttman’s lambda at 0.86 and 0.98, respectively. These values indicate that all d-Lexize versions are highly reliable.

### Validity estimates for d-Lexize

To further assess the validity of each d-Lexize version, we compared pupils’ performance in d-Lexize with their performance on two other tests, the SFR and PWR. Specifically, we calculated the correlations between the accuracy rates and reaction times of each Lexize test and those of the SFR (Experiments 1–3) and the PWR test (Experiments 2 and 3). It is important to note that both the SFR and PWR test items are relatively easy, resulting in high accuracy rates (90% or more) and minimal variation (even in the lower grades), deflating the correlations. Therefore, the reaction times in both tests are more reliable indicators of the processing effort required by the children. This is also reflected in the correlations, with the strongest associations observed between d-Lexize RTs and those of the SFR and PWR (ranging from 0.69 to 0.82), indicating excellent validity. Full correlation details are presented in Table 2. A pseudoword reading task was not included, limiting the analysis’s ability to account for variation in phonetic decoding.

**Table 2:** Correlations between accuracy rates and reaction times of d-Lexize tests with those of the SFR and the PWR tests. All bolded values are significant at the *p* < .001-level, the non-bolded values are not significant at the *p* < 0.05 level.

Variable	SFR_Acc	SFR_RT	PWR_Acc	PWR_RT
d-Lexize89_Acc	**0.33**	**− 0.45**		
d-Lexize89_RT	0.02	**0.69**		
d-Lexize55a_Acc	**0.30**	**− 0.29**	**0.33**	**− 0.13**
d-Lexize55a_RT	0.03	**0.82**	**− 0.24**	**0.71**
d-Lexize55b_Acc	**0.25**	**− 0.35**	**0.32**	**− 0.23**
d-Lexize55b_RT	0.03	**0.82**	**− 0.30**	**0.72**
d-Lexize41a_Acc	**0.43**	**− 0.46**	**0.45**	**− 0.30**
d-Lexize41a_RT	**− 0.10**	**0.79**	**− 0.27**	**0.72**
d-Lexize41b_Acc	**0.39**	**− 0.42**	**0.40**	**− 0.24**
d-Lexize41b_RT	**− 0.09**	**0.80**	**− 0.27**	**0.73**

The validity of the different d-Lexize versions was also evaluated by examining the effects of word frequency and length on accuracy and reaction times, as these factors are known to influence reading and lexical decision tasks^22,23^. Both factors were analyzed in interaction with grade, as we expected their effects to vary by grade—a pattern that emerged in most analyses. The corresponding statistics and figures illustrating these interactions are presented in the mixed-effects models section of the HTML reports. Most importantly, consistent with previous research and across all grades and experiments, lower word frequency was associated with lower accuracy and slower reaction times, while longer words led to slower reaction times, supporting the validity of all d-Lexize versions.

### Ethics declarations

The study utilized pseudonymized registry data collected initially by municipal education authorities as part of their statutory monitoring of pupils’ competences. Data collection was carried out in collaboration with the University of Turku’s TRILA research unit and in consultation with researchers to ensure the collection of information valuable for monitoring. Each municipality’s educational office independently decided whether to participate in the assessments organized by the University. Furthermore, the municipalities determined the specific grade levels that would be included in the evaluation. The primary objective of the assessments was to provide teachers and educational authorities within each municipality with information regarding pupils’ strengths and challenges in reading and mathematical skills. To facilitate the provision of feedback to teachers about their own pupils, each child was identified using a school-based login system. All experimental protocols were approved by each municipality or municipal consortium in accordance with official procedures. More than 20 municipalities participated in this collaboration. Specifically, the municipalities or municipal consortiums that approved the experimental protocols were City of Helsinki, City of Turku, City of Salo, The ‘School in Shape’ municipal consortium (‘Koulu kunnossa’ in Finnish, including the municipalities Lohja, Hanko, Inkoo, Raasepori, Karkkila, Siuntio, Vihti, Kirkkonummi, Kauniainen), the ‘Way of Doing things’ municipal consortium (‘Konsti’ in Finnish, including the municipalities Sauvo, Raisio, Parainen, Paimio, Naantali, Mynämäki, Lieto, Kaarina, Uusikaupunki. All necessary ethical approvals were obtained in advance of the study.

Due to the municipalities’ legal autonomy, the procedures for obtaining guardians’ permissions were determined locally. The research activities were conducted independently from the municipal assessments and their analyses. The research team requested permission from each municipality to utilize the assessment data for research purposes, thereby repurposing pre-existing registry data. TRILA ensured that prior to data transfer, all guardians had the opportunity to withhold their child’s pseudonymized data from research use. Each municipality independently managed the process of obtaining informed parental consent, permitting the release of data to a predefined group of researchers in a pseudonymized format. This format had excluded all direct identifiers, such as personal login ID, names, school affiliations, and municipal identifiers. The study was conducted in full compliance with the guidelines of the Finnish National Board on Research Integrity (TENK).

## Supplementary Information

Below is the link to the electronic supplementary material.

Supplementary Material 1

## Data Availability

Extensive data analysis reports and R script are available at https://osf.io/exb9s/?view_only=04dda2e11463493a8cc4bc76261d5e9c. The datasets themselves, generated during and/or analyzed during the current study are available from the corresponding author on reasonable request.
