# Supplementary material for: Gender and home language effects on vocabulary skills among school children aged 9–15 in Finland
Source: Sci Rep. 2025 Dec 29;15:44832. doi: 10.1038/s41598-025-28902-w (PMC12748919; doi:10.1038/s41598-025-28902-w)
Supplement: Supplementary file 1 — Supplementary Material 1 [file 41598_2025_28902_MOESM1_ESM.docx]

**SUPPLEMENTARY MATERIAL**

**Gender and Home Language Effects on Vocabulary Skills among School Children Aged 9 to 15 in Finland**

Raymond Bertram^1^*, Tomi Rautaoja^2^, Santeri Holopainen^2^, Tuomo Häikiö^1^, Petra Enges^2^, Jukka Hyönä^1^, Minna Lehtonen^1^, Kenneth R. Pugh^3,4^, Jay G. Rueckl^3^, Rosa Salmela^2^, Noam Siegelman^5^ & Pekka Räsänen^2,6^

^1^Department of Psychology and Speech-Language Pathology, University of Turku, Finland

^2^Turku Research Institute for Learning Analytics, University of Turku, Finland

^3^Department of Psychological Sciences, University of Connecticut, USA

^4^Yale Reading Center, Yale School of Medicine, USA

^5^Departments of Psychology and Cognitive and Brain Sciences, Hebrew University of Jerusalem, Israel

^6^Epilepsia Helsinki, Department of Pediatric Neurology, HUS Helsinki University Hospital, Finland

**Stimulus Lists (Lexize, d-Lexize89, d-Lexize55a, d-Lexize55b, d-Lexize41a, d-Lexize41b)**

**Supplementary Table 1**. All words (with English translations, ordered by increasing frequency) and pseudowords as used in different versions of Lexize.

| **Idnr** | **Item** | **Translation** | **Lex^a^** | **PoS^b^** | **L102^c^** | **dL89^d^** | **dL55a^e^** | **dL55b^f^** | **dL41a^g^** | **dL41b^h^** |
| --- | --- | --- | --- | --- | --- | --- | --- | --- | --- | --- |
| 1 | juhta | beast of burden | word | n | 1 | 1 |  | 1 |  |  |
| 2 | itara | stingy | word | a | 1 |  |  |  |  |  |
| 3 | kovera | concave | word | a | 1 |  |  |  |  |  |
| 4 | vauhko | raving | word | a | 1 | 1 | 1 |  |  | 1 |
| 5 | heltta | wattle | word | n | 1 | 1 | 1 | 1 |  | 1 |
| 6 | kiulu | pail | word | n | 1 | 1 | 1 | 1 | 1 |  |
| 7 | känsä | callus | word | n | 1 | 1 | 1 |  |  | 1 |
| 8 | kolttu | dress | word | n | 1 | 1 | 1 | 1 |  |  |
| 9 | mankua | to whine | word | v | 1 | 1 | 1 |  | 1 |  |
| 10 | kekäle | ember | word | n | 1 | 1 |  | 1 |  | 1 |
| 11 | vohkia | to nick | word | v | 1 | 1 | 1 |  |  | 1 |
| 12 | hökkeli | shack | word | n | 1 | 1 | 1 |  | 1 |  |
| 13 | houre | delirium | word | n | 1 |  |  |  |  |  |
| 14 | nyplätä | to make lace | word | v | 1 | 1 |  | 1 | 1 |  |
| 15 | uuhi | ewe | word | n | 1 |  |  |  |  |  |
| 16 | kieppi | coil | word | n | 1 |  |  |  |  |  |
| 17 | vouti | magistrate | word | n | 1 |  |  |  |  |  |
| 18 | haikara | stork | word | n | 1 | 1 | 1 |  | 1 |  |
| 19 | luikkia | to slink | word | v | 1 | 1 |  | 1 |  | 1 |
| 20 | karkelo | frolic | word | n | 1 | 1 | 1 |  |  | 1 |
| 21 | usva | haze | word | n | 1 | 1 |  | 1 |  | 1 |
| 22 | aihio | work in progress | word | n | 1 |  |  |  |  |  |
| 23 | rihkama | bauble | word | n | 1 | 1 | 1 | 1 |  | 1 |
| 24 | metku | trick | word | n | 1 | 1 | 1 |  |  | 1 |
| 25 | pytty | wooden tub | word | n | 1 | 1 |  | 1 | 1 |  |
| 26 | navakka | brisk | word | a | 1 | 1 |  | 1 | 1 |  |
| 27 | hihna | belt | word | n | 1 | 1 | 1 |  |  | 1 |
| 28 | veruke | pretext | word | n | 1 | 1 |  | 1 |  | 1 |
| 29 | keidas | oasis | word | n | 1 | 1 |  | 1 | 1 |  |
| 30 | kiikastaa | to be the problem | word | v | 1 | 1 |  | 1 | 1 |  |
| 31 | holvi | vault | word | n | 1 | 1 |  | 1 | 1 | 1 |
| 32 | hulina | razzmatazz | word | n | 1 | 1 | 1 |  | 1 |  |
| 33 | mahla | sap | word | n | 1 | 1 |  | 1 | 1 |  |
| 34 | huokea | inexpensive | word | a | 1 | 1 |  | 1 | 1 |  |
| 35 | rahvas | common people | word | n | 1 | 1 | 1 | 1 |  |  |
| 36 | uuttera | diligent | word | a | 1 | 1 | 1 |  | 1 | 1 |
| 37 | toukka | maggot | word | n | 1 | 1 | 1 | 1 | 1 |  |
| 38 | napa | belly button | word | n | 1 | 1 | 1 |  |  | 1 |
| 39 | seula | screen | word | n | 1 | 1 | 1 |  | 1 |  |
| 40 | keko | hill | word | n | 1 | 1 |  | 1 |  | 1 |
| 41 | pisara | drop | word | n | 1 | 1 |  | 1 |  |  |
| 42 | pitsi | lace | word | n | 1 | 1 |  | 1 | 1 | 1 |
| 43 | nokkela | clever | word | a | 1 | 1 | 1 | 1 | 1 |  |
| 44 | pylväs | pillar | word | n | 1 | 1 | 1 | 1 | 1 |  |
| 45 | kohtu | uterus | word | n | 1 | 1 |  | 1 |  |  |
| 46 | hauras | fragile | word | a | 1 | 1 | 1 |  |  | 1 |
| 47 | jyvä | grain | word | n | 1 | 1 | 1 |  | 1 |  |
| 48 | ahne | greedy | word | a | 1 | 1 | 1 |  |  | 1 |
| 49 | kuopus | youngest child | word | n | 1 | 1 | 1 | 1 |  | 1 |
| 50 | tyrkyttää | impose | word | v | 1 | 1 | 1 |  |  |  |
| 51 | parvi | loft | word | n | 1 | 1 | 1 | 1 |  |  |
| 52 | loukku | trap | word | n | 1 | 1 | 1 |  | 1 |  |
| 53 | purje | sail | word | n | 1 |  |  |  |  |  |
| 54 | laimea | diluted | word | a | 1 | 1 | 1 | 1 |  | 1 |
| 55 | siivu | slice | word | n | 1 | 1 | 1 | 1 |  | 1 |
| 56 | nuotio | campfire | word | n | 1 | 1 |  | 1 |  |  |
| 57 | kainalo | armpit | word | n | 1 | 1 | 1 |  | 1 |  |
| 58 | hauki | pike | word | n | 1 | 1 | 1 |  |  |  |
| 59 | taikina | dough | word | n | 1 | 1 |  | 1 |  | 1 |
| 60 | suppea | narrow | word | a | 1 |  |  |  |  |  |
| 61 | navetta | barn | word | n | 1 | 1 |  | 1 |  | 1 |
| 62 | jäykkä | stiff | word | a | 1 | 1 |  | 1 | 1 |  |
| 63 | sukeltaa | dive | word | v | 1 | 1 | 1 |  |  |  |
| 64 | rotu | race | word | n | 1 | 1 |  | 1 | 1 |  |
| 65 | haju | smell | word | n | 1 | 1 | 1 |  |  | 1 |
| 66 | liekki | flame | word | n | 1 | 1 | 1 | 1 | 1 |  |
| 67 | kosto | revenge | word | n | 1 | 1 | 1 |  | 1 |  |
| 68 | ruuhka | traffic jam | word | n | 1 | 1 |  | 1 |  | 1 |
| 69 | hahna | NA | pseudoword | NA | 1 | 1 | 1 |  | 1 |  |
| 70 | halkahtaa | NA | pseudoword | NA | 1 | 1 | 1 | 1 | 1 |  |
| 71 | hilmo | NA | pseudoword | NA | 1 | 1 | 1 |  | 1 |  |
| 72 | hirsto | NA | pseudoword | NA | 1 | 1 | 1 | 1 | 1 |  |
| 73 | jyhmys | NA | pseudoword | NA | 1 | 1 | 1 |  | 1 |  |
| 74 | kahmea | NA | pseudoword | NA | 1 | 1 | 1 | 1 |  | 1 |
| 75 | kammu | NA | pseudoword | NA | 1 | 1 |  | 1 |  | 1 |
| 76 | kapottaa | NA | pseudoword | NA | 1 | 1 | 1 | 1 |  | 1 |
| 77 | kolje | NA | pseudoword | NA | 1 |  |  |  |  |  |
| 78 | linniö | NA | pseudoword | NA | 1 | 1 | 1 |  | 1 |  |
| 79 | lukkara | NA | pseudoword | NA | 1 | 1 |  | 1 |  | 1 |
| 80 | meltainen | NA | pseudoword | NA | 1 | 1 | 1 |  | 1 |  |
| 81 | muihko | NA | pseudoword | NA | 1 | 1 | 1 |  | 1 |  |
| 82 | noikka | NA | pseudoword | NA | 1 | 1 |  | 1 |  | 1 |
| 83 | nökkelö | NA | pseudoword | NA | 1 | 1 |  | 1 |  | 1 |
| 84 | olvota | NA | pseudoword | NA | 1 | 1 | 1 |  | 1 |  |
| 85 | ouvaista | NA | pseudoword | NA | 1 | 1 | 1 |  | 1 |  |
| 86 | paaskua | NA | pseudoword | NA | 1 |  |  |  |  |  |
| 87 | pilja | NA | pseudoword | NA | 1 | 1 |  | 1 |  | 1 |
| 88 | pylökkö | NA | pseudoword | NA | 1 | 1 | 1 |  | 1 |  |
| 89 | raipulo | NA | pseudoword | NA | 1 | 1 | 1 | 1 |  | 1 |
| 90 | ruihka | NA | pseudoword | NA | 1 | 1 |  | 1 |  | 1 |
| 91 | serma | NA | pseudoword | NA | 1 |  |  |  |  |  |
| 92 | sihu | NA | pseudoword | NA | 1 |  |  |  |  |  |
| 93 | siikko | NA | pseudoword | NA | 1 | 1 | 1 |  | 1 |  |
| 94 | soilu | NA | pseudoword | NA | 1 | 1 | 1 | 1 | 1 |  |
| 95 | taipo | NA | pseudoword | NA | 1 | 1 |  | 1 |  | 1 |
| 96 | tihmontaa | NA | pseudoword | NA | 1 | 1 |  | 1 |  | 1 |
| 97 | tolju | NA | pseudoword | NA | 1 | 1 | 1 | 1 |  | 1 |
| 98 | tulvus | NA | pseudoword | NA | 1 | 1 |  | 1 |  | 1 |
| 99 | tämsä | NA | pseudoword | NA | 1 | 1 | 1 |  | 1 |  |
| 100 | ukara | NA | pseudoword | NA | 1 | 1 |  | 1 |  | 1 |
| 101 | vahnia | NA | pseudoword | NA | 1 | 1 |  | 1 |  | 1 |
| 102 | vikku | NA | pseudoword | NA | 1 | 1 | 1 | 1 | 1 |  |

a. Lexicality; b. Part of Speech; c. Lexize; d. d-Lexize89; e. d-Lexize55a; f.; d-Lexize55b g. d-Lexize41a; h. d-Lexize41b
